# Identifying high‐density areas of oysters using species distribution modeling: Lessons for conservation of the native *Ostrea edulis* and management of the invasive *Magallana* (*Crassostrea*) *gigas* in Sweden

**DOI:** 10.1002/ece3.7451

**Published:** 2021-03-22

**Authors:** Per Bergström, Linnea Thorngren, Åsa Strand, Mats Lindegarth

**Affiliations:** ^1^ Department of Marine Sciences –Tjärnö University of Gothenburg Tjärnö Sweden; ^2^ IVL Swedish Environmental Research Institute Fiskebäckskil Sweden

**Keywords:** conservation, distribution, ensemble modeling, management, oyster, protection

## Abstract

**Aim:**

Understanding spatial patterns of the distribution of adult native oyster, *Ostrea edulis*, and the invasive *Magallana (Crassostrea) gigas* is important for management of these populations. The aim of this study was to use ensemble SDM’s to (a) identify and predict conservation hotspots, (b) assess the current level of protection for *O. edulis,* and (c) quantify the amount of overlap between the two species where interactions with *M. gigas* are most likely.

**Location:**

Skagerrak, Sweden.

**Methods:**

We used data collected by video at depths from 0.5 to 10 m in 436 sites. Models of occurrence and densities >1 m^−2^ were fitted and assessed using ensemble methods (“biomod2” package). Models of high‐density hotspots were used to predict, map, and quantify areal extent of the species in order to assess the degree of overlap with protected areas and the potential for interactions between the two species.

**Results:**

Both species were widely distributed in the region. Observations of high‐density habitats, mainly occurring at depths of ≈3 and 0.5 m for *O. edulis* and *M. gigas*, respectively, were found in 4% and 2% of the sites. Models provided useful predictions for both species (AUC = 0.85–0.99; sensitivity = 0.74–1.0; specificity = 0.72–0.97). High‐density areas occupy roughly 15 km^2^ each with substantial overlap between species. 50% of these are protected only by fisheries regulations, 44% are found in Natura 2000 reserves and 6% of the predicted *O. edulis* enjoys protection in a national park.

**Main conclusions:**

Data collection by video in combination with SDM’s provides a realistic approach for large‐scale quantification of spatial patterns of marine population and habitats. *O. edulis* and *M. gigas* are common in the area, but a large proportion of the most valuable *O. edulis* habitats are not found in protected areas. The overlap between species suggests that efforts to manage the invasive *M. gigas* need to be integrated with management actions to conserve the native *O. edulis*.

## Graphical abstract

Understanding spatial patterns of the native oyster, *Ostrea edulis*, and the invasive *Magallana gigas* is important for management of these populations. We used data collected by video at depths from 0.5 to 10 m in 436 sites. Models of occurrence and densities > 1 m^−2^ were fitted and assessed using ensemble methods. Data collection by video in combination with SDM’s provides a realistic approach for large‐scale quantification of spatial patterns of marine population and habitats.

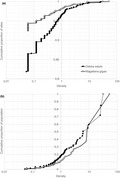

## 1 INTRODUCTION

Many important structural and functional components of benthic marine ecosystems, such as mussel‐ and oyster beds, seagrass meadows and coral reefs, are currently in global decline and threatened by local extinction and fragmentation (Airoldi & Beck, 2007; Beck et al., 2011; Harwell et al., 2011). The reasons for these impacts are complex and varies among regions and habitats, but it is clear that increasing and more diverse human pressures contributes substantially to habitat loss and deterioration and that the current status do not reflect the true ecological potential of these habitats (Costello, 2014; Halpern et al., 2008). In response to this, protection and sustainable management of habitats and their associated ecosystem services has received increasing attention in policies developed around the world (CEQ, 2010; European Commission, 2010, 2014).

One habitat of special concern in Europe is the biogenic reefs (OSPAR framework (Council of the European Union, 1998), the Marine Strategy Framework Directive (European Commission, 2008), the Habitat Directive (Council of the European Union, 1992). Biogenic reefs are aggregations of living or dead organisms, for example, bivalves, worms, or corals, which act as “ecological engineers” by creating three‐dimensional habitat for associated species in benthic ecosystems across the globe (Jones et al., 1994). This contributes to increased ecosystem resilience and supports a wide range of ecosystem services (Fletcher et al., 2011). Bivalve reefs also contribute to regulating services (see Zu Ermgassen et al., 2020 for an extensive review of services provided by shellfish), such as having the potential to improve water quality (Grizzle et al., 2018; Kreeger et al., 2018; Parker & Bricker, 2020), as well as cultural and provisioning ecosystem services, for example, through fisheries and aquaculture (Grabowski & Peterson, 2007; Laugen et al., 2015; Ruesink et al., 2005). Despite recognition of its importance and the following development of protective policies, bivalves such as the European flat oyster (*Ostrea edulis* Linnaeus, 1758) has suffered substantial population decline. A few centuries ago, they formed extensive beds and were an important part of the ecosystems along the European and Mediterranean coasts (Beck et al., 2011 and references therein). This situation has now changed due to overexploitation, habitat destruction, and pathogens; these habitats are presently considered as one of the most endangered in European waters (Airoldi & Beck, 2007; Beck et al., 2011).

Even though the core distribution of *O. edulis* is found along the European Atlantic coast and into the Mediterranean, it is well known that the species also occur and may form dense beds in Swedish parts of the Skagerrak. Until recently, however, the prevalence, abundance, and environmental preferences of this population have been poorly studied and not quantitatively documented. Thorngren et al., (2019) concluded that the abundance of the Swedish population was in fact substantial in a European context. Furthermore, the study confirmed recent observations that the invasive oyster *Magallana (Crassostrea) gigas* (Thunberg, 1793), hereafter *M. gigas*, is now established along the Swedish west coast (Faust et al., 2017; Laugen et al., 2015; Mortensen et al., 2016; Strand et al., 2012), potentially in habitats and locations where *O. edulis* thrives (Laugen et al., 2015). These results suggest that management of *O. edulis* in the region can benefit greatly from a better understanding of factors determining the spatial distribution of high‐density beds and niche overlap between the two species. The latter is particularly interesting from a management point of view because the native *O. edulis* is protected under OSPAR and European habitats directive (Council of the European Union, 1992, 1998), while the alien *M. gigas* is considered invasive and potentially harmful (Zwerschke et al., 2018). Nevertheless, the most important legal instrument directly regulating the exploitation of oysters, the Swedish “fisheries‐law”, does not distinguish between the two species and fishing rights are reserved to the landowner (§ 9, SFS 1993:787). Similarly, in protected areas along the coast (e.g., Kosterhavet National Park (KNP) and numerous Natura 2000 reserves), both species enjoy the same level of protection, which means that they cannot be collected without landowner permits. The landowner of national parks in Sweden is always the state and in theory this means that both species enjoy full protection in the KNP. However, the full implementation and compliance to these regulations cannot always be taken for granted.

Understanding patterns and processes associated with high‐density areas of the native *O. edulis*, the invasive *M. gigas* and the interactions between the two are important components in future sustainable management of these populations and the biogenic reefs that they provide. Therefore, the aim of this study was to assess and model the distributions of the two species in coastal Skagerrak. We used data from an extensive field study in combination with species distribution models (SDM) to identify and predict conservation hotspots, that is, areas and conditions most likely to have high densities of the native *O. edulis*, and areas where competitive interactions from high densities of the invasive *M. gigas* is most likely. Furthermore, we used the predicted distributions to analyze the degree to which these populations and reefs are encompassed by protected areas. These analyses will provide new knowledge on the spatial distribution, environmental requirements, and potential interactions of the two species and shed further light on challenges associated with management of these valuable habitats.

## 2 METHODS

### 2.1 Study area and data collection

**FIGURE 1:**
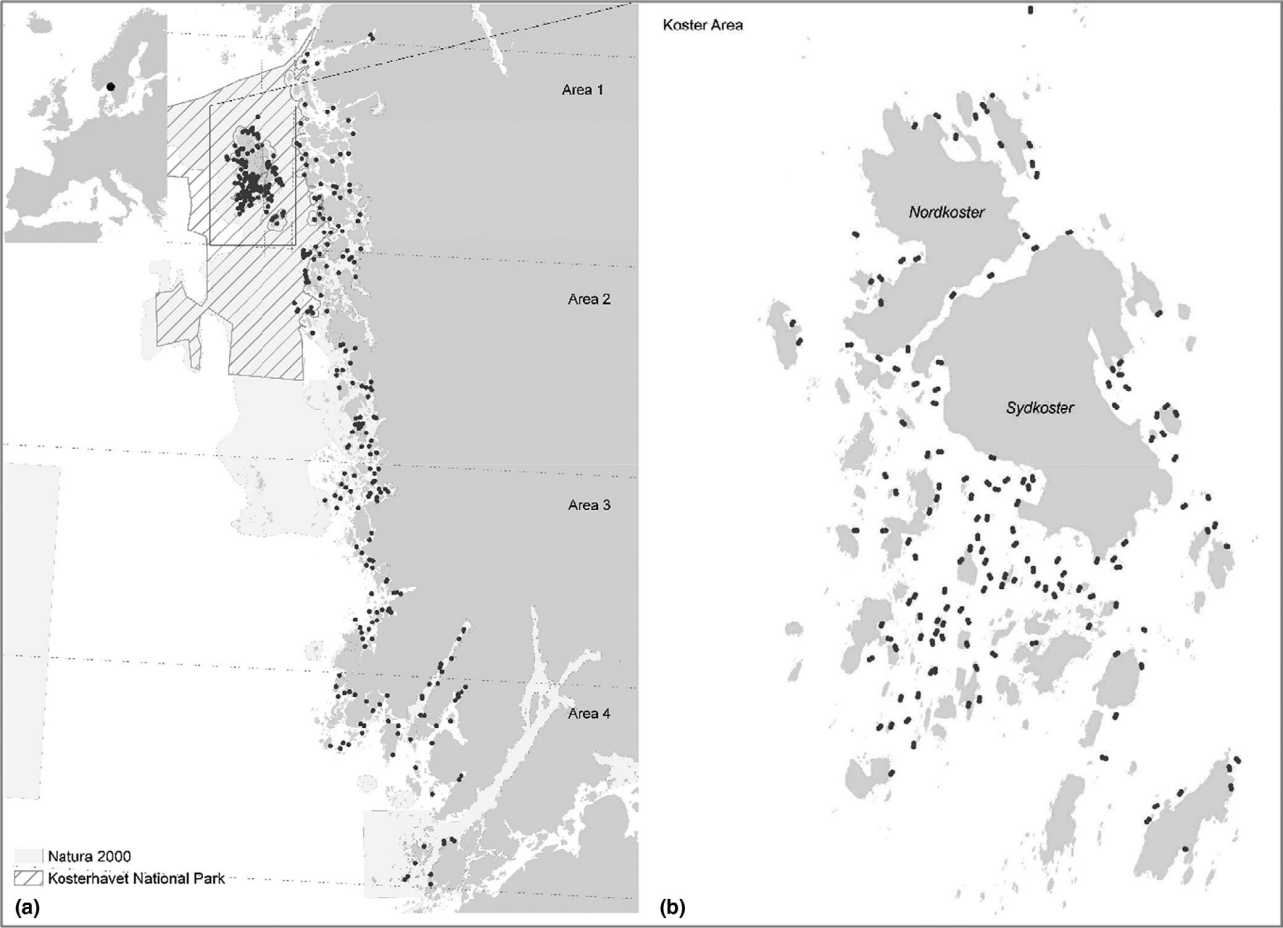
Study site and sample distribution. (a) Overview of sampled areas and (b) close‐up of sampled locations around the more densely sampled Koster archipelago situated within the Kosterhavet National Park

The study was carried out along the part of the Swedish west coast where the majority of the Swedish native flat oyster population occurs (Figure 1). This coastal area is characterized by small tidal range (±0.2 m), fluctuating salinities (commonly 20–30 psu in surface water), naturally fragmented habitats, and a wide range of wave exposure conditions.

Data on oyster abundance from two studies performed in 2013 and 2014 were used, one study covering the whole stretch of the coast mentioned above and one concentrated to the KNP (Lindegarth et al., 2014; Thorngren et al., 2019). Both studies used identical field protocols based on towed video transects and subsequent image analyses of oyster density and habitat characteristics (see Thorngren et al., 2017 for detailed description of methods). Survey sites were selected by randomized, stratified sampling resulting in 436 sites across the region (Area 1–4 and Koster, Figure 1) and three depth intervals (0.5–3, 3–6, and 6–10 m).

Four predictor variables reflecting physical and chemical aspects (depth, exposure, salinity, and bottom substrate) known to affect the composition of benthic organisms and habitats in general, and the distribution of oysters in particular, were used (Lindegarth et al., 2014; Snickars et al., 2014). Observations of depth were obtained from field measurements, and bottom substrates were estimated from filmed transects. Wave exposure was extracted from a modeled national map of exposure (Swedish Environmental Protection Agency, 2006) and subsequently transformed into depth‐attenuated exposure according to Bekkby et al. (2008). Minimum salinity for each site was obtained from an interpolated GIS layer (Bergström, unpublished; based on >20,000 measurements from 1988 to 2018 in Skagerrak and Kattegat retrieved from ICES [https://www.ices.dk/marine‐data/dataset‐collections/Pages/default.aspx]).

### 2.2 Ensemble modeling of oysters

An ensemble modeling approach was used to model and predict the spatial distributions of *Ostrea edulis* and *Magallana gigas*. Both presence versus absence of single oysters, as well as of high‐density areas (defined as ≥1 oyster m^−2^) were modeled. Empirical models were implemented in the R software (R Core Team, 2017) using the “BIOMOD2” packages (Thuiller et al., 2016). Ensemble models were based on nine different methods using default parameters: Random Forest, Generalized Boosting Models, Multivariate Regression Splines, Generalized Linear Models, Surface Range Envelope, Artificial Neural Networks, Classification Tree Analysis, Generalized Additive Model, and Flexible Discriminant Analysis. Models were assessed using a 100‐fold cross‐validation and randomly splitting the data into training (70%) and test data (30%) for model calibration and testing, respectively, allowing for evaluation of model accuracy and predictive performance using internal and external validation. Performance of models was assessed using area under receiver operating curve (AUC), sensitivity, and specificity (Pearson, 2007). Only models above a critical AUC value of 0.7 were included and combined into the final ensemble models to ensure the inclusion of only statistically reliable models (Hosmer & Lemeshow, 2000). Ensemble models were evaluated using the same performance statistics as individual models (i.e., AUC, sensitivity, and specificity). The functional relationships between the environmental predictors (depth, exposure, salinity, and bottom substrate) and the probability occurrence were further explored using analyses of variable importance and partial dependence plots.

The purpose of the models was twofold. Models were fitted and tested to assess the importance and predictive power of all four environmental variables. Secondly, models were used to create full‐covering spatial predictions in order to assess the distribution of high‐density oyster areas, that is, densities of >1 m^−2^. In contrast to depth, exposure, and salinity, data on bottom substrate were obtained from the videos and as a comprehensive map of substrate information in the region is not available, only the models without substrate were used for the spatial predictions (i.e., predict of distribution in nonsampled areas).

### 2.3 Spatial prediction and protection of high‐density areas

The fitted models of high‐density occurrence for the two species were applied to high‐resolution (15 × 15 m) raster layers of depth, depth‐attenuated exposure, and minimum salinity. This generated predictions of probabilities of occurrence of high‐density areas and maps of occurrence were created using the default cutoff values (calculated to maximize sensitivity and specificity). The maps were used to assess the areal extent and level of protection of essential oyster habitats. Thus, predictive maps of high‐density areas of *O. edulis* and *M. gigas* were used (a) to estimate the total area and (b) to estimate the overlap among species and with GIS maps of the KNP and Swedish Natura 2000 areas (http://gpt.vic‐metria.nu/data/land/SCI_Rikstackande.zip).

The predicted extent of high‐density areas was compared with the extent inferred from observed frequencies within strata. The representative data from the field studies were used in combination with GIS information on the areal extent of depth strata within the five areas (Figure 1). Because random, representative samples were allocated separately to depth strata in the two studies, frequencies of high‐density areas (${\hat{p}}_{\text{st}}$), and variances ($s_{\text{st}}^{2}$) were calculated separately for depth strata within each study before they were combined into weighted estimates of (${\hat{p}}_{W}$) and variances $V({\hat{p}}_{W})$ according to procedures for stratified sampling described in Cochran (1977):

(1) $${\hat{p}}_{W}=\sum_{\text{St}}W_{\text{st}}*{\hat{p}}_{\text{st}}$$

and

(2) $$V\left({\hat{p}}_{W}\right)=\sum_{\text{St}}\frac{W_{\text{st}}^{2}*s_{\text{st}}^{2}}{n_{\text{st}}}$$

where the variance within a stratum, $s_{\text{st}}^{2}$, is approximated as ${\hat{p}}_{\text{st}}*\left(1‐{\hat{p}}_{\text{st}}\right)/n_{\text{st}}$, $W_{\text{st}}$ is the weight (areal proportion) of a stratum and $n_{\text{st}}$ is the number of samples within a stratum. Finally, the total extent of observed high‐density areas in the region and its associated error was calculated as:

(3) $${\hat{p}}_{W}*{\text{Area}}_{\text{Total}}\pm \sqrt{V(p_{W})}*{\text{Area}}_{\text{Total}}$$

where ${\text{Area}}_{\text{Total}}$ is the total extent of the area investigated with representative samples.

## 3 RESULTS

### 3.1 Prevalence of occurrence and high‐density occurrence

**FIGURE 2:**
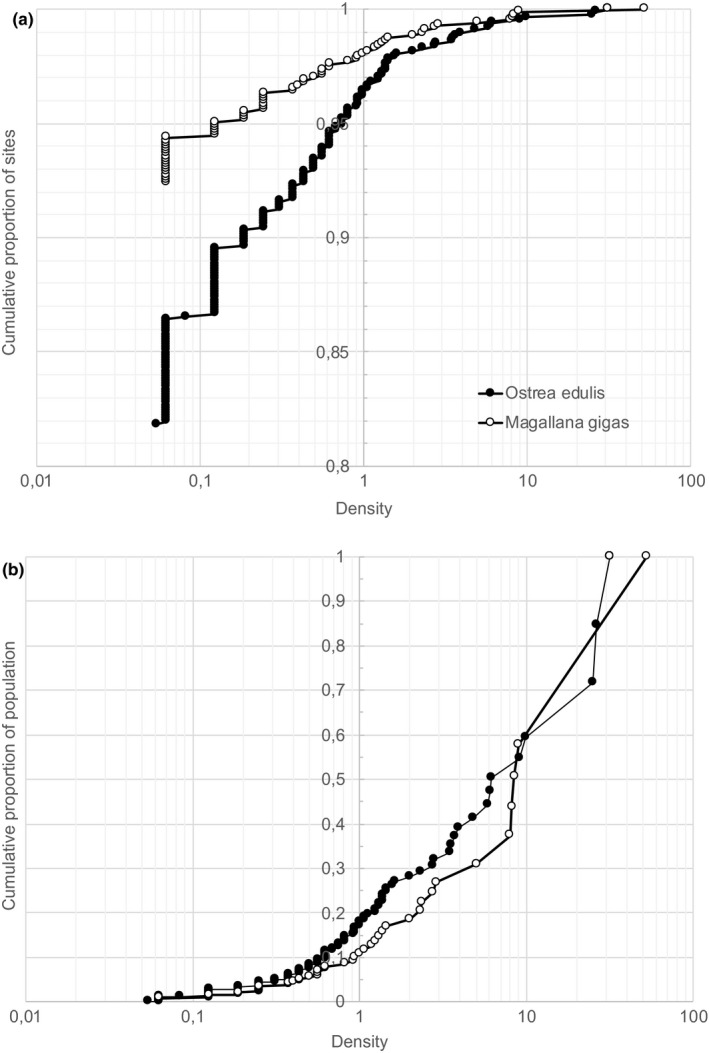
Cumulative proportion of (a) sites and (b) relative population size as function of density (living individuals m^−2^) in samples from the Swedish west coast. Note the logarithmic *x*‐axes and that the *y*‐axis are truncated in (a). Both plots show only sites containing oysters. *O. edulis* and *M. gigas* were only found in 19% and 8% of the sampled sites (see Figure 2a)

*Ostrea edulis* and *Magallana gigas* were found in 159 and 67 transects (18% and 8%) of all transects, respectively (Table 1; Figure 2a). One striking observation is that all sites colonized by *M. gigas* also contained *O. edulis*. The largest densities observed for *O. edulis* and *M. gigas* were 31.6 and 52.5 individuals m^−2^, respectively. Although there were smaller patches with large densities, average densities in the transects were ≥1 m^−2^ only in 34 and 18 transects (4% and 2% of the all transects and 21% and 27% of transects containing oysters). The largest frequencies of high‐density sites were found in areas 2 and 3 for both species.

**TABLE 1:** Prevalence (%) of oysters (present) and of high‐density (≥1 oyster m^‐2^) sites for *Ostrea edulis* and *Magallana gigas* in different areas

Species	Response	Area 1	Area 2	Area 3	Area 4	Koster	Total
*O. edulis*	Presence	16.0	26.0	17.1	11.7	18.3	18.3
	High	3.8	10.4	2.1	0.8	2.9	3.9
*M. gigas*	Presence	8.5	15.6	16.4	5.8	1.1	7.7
	High	3.8	5.2	3.6	0.8	0	2.1
Both species	Presence	8.5	15.4	16.4	5.8	1.1	7.7

Despite the low number of high‐density sites, the contribution of these few sites to the overall population was very important for both species (Figure 2b). Assuming a representative frequency distribution and plotting the cumulative distribution of abundance of the two species, it was observed that roughly 85% of the *O. edulis* and ~90% of the *M. gigas* populations were found at densities ≥1 individuals m^−2^ and that ~40% of both populations are found at densities of ten oysters per m^−2^ or higher (Figure 2b). Apart from revealing the great importance of a few sites where oysters were particularly abundant, this information was also used to provide a definition of high‐density areas. Despite the fact that “oyster beds” for conservation purposes often are defined as having densities ≥5 individuals m^−2^ (Haelters & Kerckhof, 2009), a more inclusive definition (i.e., ≥1 m^−2^) was used in this study, thereby involving <5% of the sites but 85%–90% of the population of the two species.

### 3.2 Fitting species distribution models

Ensemble models performed generally well for *O. edulis* using depth, exposure, and salinity as explanatory variables (AUC 0.85–0.95), but excellent (AUC 0.9–1.0) with the addition of sediment properties (percentage gravel, Table 2). A similar pattern was observed in the *M. gigas* models were the AUC increased slightly when including substrate (Table 2). Similarly, the sensitivity and specificity of the models increased when including substrate as an explanatory variable. Generally, the high‐density models performed better than the presence models irrespectively of the inclusion of substrate, and high‐density models were generally better at finding true positives than true negatives, that is, were better at finding the places with oysters (presence or high density) than sites without (Table 2). This was particularly true for *O. edulis* where the specificity was ≈0.8 for occurrence and high‐density models.

**TABLE 2:** Model performance of ensemble models for *Ostrea edulis* and *Magallana gigas* with substrate (+S) and without substrate (−S) properties using external validation on test data

Species	Response	AUC		Sensitivity (“true positives”)		Specificity (“true negatives”)		Cutoff	
		+S	−S	+S	−S	+S	−S	+S	−S
*O. edulis*	Presence	0.88	0.85	0.84	0.74	0.76	0.77	0.27	0.32
	High	0.97	**0.95**	0.93	**0.96**	0.93	**0.82**	0.14	**0.10**
*M. gigas*	Presence	0.94	0.91	0.91	0.96	0.86	0.72	0.16	0.12
	High	0.99	**0.99**	1.00	**1.00**	0.96	**0.97**	0.13	**0.14**

Note

Bold numbers are statistics representing models used for predictions. Presence = occurrence of oyster, High ≥ 1 oyster m^−2^.

Analyses of variable importance suggested that substrate (i.e., gravel) were the most important factor for *O. edulis* (0.49–0.52) followed by depth (0.28–0.47 in the ensemble modeling; Table 3). For *M. gigas,* the pattern was different with depth being most important (0.57–0.65; Table 3). For models without substrate, depth was the most important predictor (0.63–0.79) but also exposure and salinity contributed somewhat (Table 3).

**TABLE 3:** Variable importance of predictors in ensemble models with substrate (+S) and without (−S) properties. Presence = occurrence of oyster, High ≥ 1 oyster m^−2^

Species	Response	% Gravel	Exposure		Depth		Minimum salinity	
		+S	+S	−S	+S	−S	+S	−S
*O. edulis*	Presence	0.52	0.12	0.21	0.28	0.68	0.08	0.21
	High	0.49	0.17	0.29	0.47	0.69	0.06	0.18
*M. gigas*	Presence	0.16	0.23	0.26	0.65	0.79	0.10	0.12
	High	0.16	0.26	0.32	0.57	0.63	0.36	0.46

**FIGURE 3:**
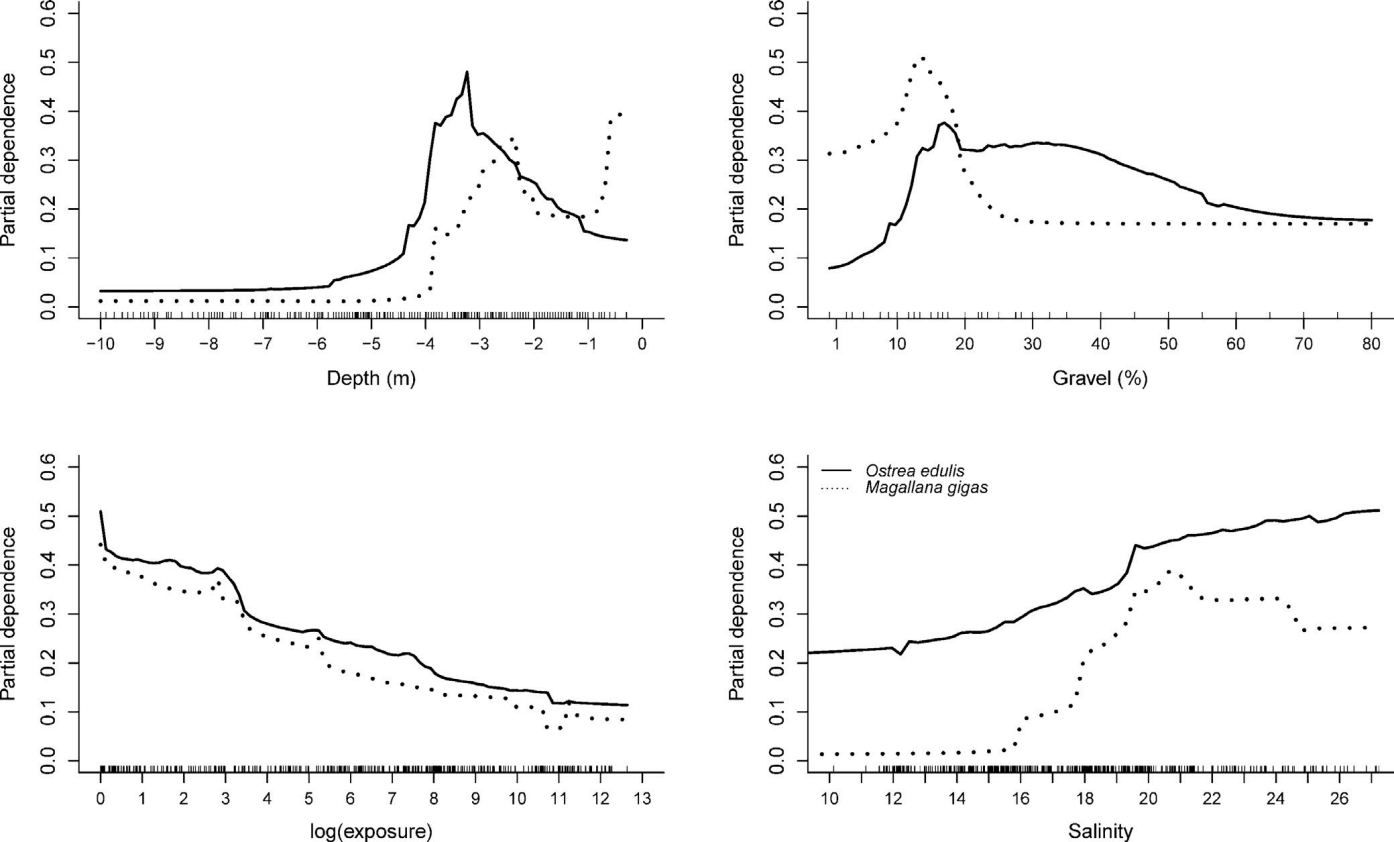
Partial dependence plots for the four predictors for the occurrence of *Ostrea edulis* and *Magallana gigas* high densities. Dashed lines = *M. gigas*, Solid lines = *O. edulis*. Observations marked as black tics on *x*‐axis

The functional relationship between the environmental predictors and the probability of high‐density occurrences of oysters showed a peak for *O. edulis* around 3–3.5 m depth with decreasing probability both toward the surface and down to 5 m, below which the partial dependence was almost zero (Figure 3). The pattern was similar for *M. gigas* although with an increasingly higher partial dependence at shallow depths (0.5 m). The partial dependence of *O. edulis* peaked around 15% gravel in the sediment after which it decreased and remained stable toward higher percentages (Figure 3). The partial dependence of *M. gigas* increased with increasing gravel content up to ~15% and then remained stable toward higher percentages (Figure 3). Furthermore, the probability of occurrence of high‐density areas of both *O. edulis* and *M. gigas* showed a tendency to decrease with increasing level of depth‐attenuated exposure and to increase with increasing minimum salinity, *O. edulis* in the range of 15–21 psu after which it slowly decreases while *M. gigas* increased continuously in the whole measured range.

### 3.3 Spatial prediction of high‐density areas

**FIGURE 4:**
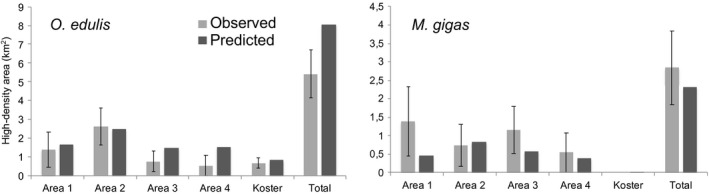
Observed and predicted extent of high‐density areas within the five subareas in the surveyed region. (±*SE*) ■ = predicted area

In order to estimate the overall areal extent and to map local occurrences of population hotspots and likely locations of biogenic reefs, we used models of high‐density occurrence to predict the distributions of both species. A total of 15 km^2^ of high‐density (>1 ind. M^−2^) occurrences of *O. edulis* areas was predicted in the investigated region (Table 4). Overall this amounts to ~10% of the total area included in the study. Approximately 25% of this area (~3.75 km^2^) contained densities exceeding 5 individuals m^−2^ (corresponding to the OSPAR definition on “oyster beds”). Although both model and observations are subject to uncertainties, it appears that the model performed reasonably well obtaining roughly the same areal extent in the different areas compared with observations (Figure 4). In three of the areas, estimated errors of observed data overlap the predicted extent of high‐density areas, while in the two southern areas 3 and 4, the model appears to overestimate the extent. This also means that the total predicted area is 48% larger than the observed. For *M. gigas,* the total area was similar to that of *O. edulis*, but predictions appeared to underestimate the distribution of high‐density areas compared with observations in area 1 and 3 (Figure 4). Both predictions and observations show that the distribution of oysters in the Koster area is different from the northern coastal areas 1 and 2, with lower frequencies of high‐density areas of the oyster species at Koster.

**TABLE 4:** Total area extent (km^2^) and per cent of predicted high‐density (≥1 oyster m^−2^) areas of *Ostrea edulis*, *Magallana gigas,* and their overlap with and without different levels of nature protection

Species	Total area	Estimate	National Park	Natura 2000	No protection
*O. edulis*	14.97	Area	0.89	6.64	7.44
		%	6	44	50
*M. gigas*	14.52	Area	0.50	7.69	6.34
		%	3	54	43
Both	7.68	Area	0.27	4.33	3.09
		%	4	56	40

Note

The total area included in the study was 121 km^2^. No protection = no other protection than the one implemented by the landowners fishing rights.

### 3.4 Levels of protection

Spatial predictions suggest that ~50% of the high‐density areas of *O. edulis* were located in areas without any other protection than that implemented by the landowners fishing rights (Table 4). Six % of the high‐density areas were covered by the strictest level of protection by being included in the KNP. The remaining 44% were found in Natura 2000 areas. The actual level of protection achieved by this type of MPA is complex. Landowners right to fishing still applies to these areas and may be entitled to compensation by the state if this right is retracted. This had to date not been applied but such measures are under discussion. In terms of other activities, for example, exploitation or construction work, Natura 2000 offer significant protection if the oyster bed exceeds 10% oyster coverage.

As explained above, the Swedish legal system does not distinguish between the two species and thus the invasive *M. gigas* in theory enjoys the same level of protection as *O. edulis*. The total high‐density area of *M. gigas* was predicted to be ~15 km^2^ (Table 4). Both models and observations showed that there were few high‐density areas in the KNP (3%), while 54% was found in Natura 2000 areas. Thus, at this stage it appears that the potential for interactions between the two species do not occur in the national park to any larger degree, while the potential is a little stronger in Natura 2000 areas. In fact, ~65% of the areas predicted to contain high densities of *O. edulis* (~6.6 km^2^) in Natura 2000 areas are also likely to have high densities of *M. gigas* (~4.3 km^2^). The corresponding proportion in the KNP is 30%.

## 4 DISCUSSION

Recent analyses of the population of the European flat oyster, *Ostrea edulis,* in the Swedish Skagerrak area have shown that, despite its peripheral location in relation to the species’ biogeographic distribution, the densities and population size in this region are substantial compared with other European locations (Thorngren et al., 2019; for other examples of remaining strong populations of *O. edulis* see Allison et al., 2020; Lown et al., 2020). These analyses also demonstrated strong spatial variability in abundance within the area and, importantly, that rare high‐density oyster beds contribute disproportionally to the total population size. In this study, we provide further support for conservation and sustainable management of oysters, particularly of the valuable high‐density beds. This is achieved by modeling the spatial distribution of oysters and analyzing the factors driving the patterns of *O. edulis*, including its overlap with the invasive Pacific oyster, *Magallana gigas*. These analyses provide new insights about (a) the ecology of and interactions among the two species in the Skagerrak region, (b) the utility of SDM as a tool for management of vulnerable species and habitats, and (c) the status of current protective measures of the Swedish oyster populations.

Several new ecological insights can be derived about the Swedish oyster populations from the observations and models presented here. Both species are widespread at depths shallower than 5 m, and the distribution is closely related to environmental factors in the area. The bottom substrate, represented by % cover of gravelly substrates, was the most important predictor for *O. edulis*. The probability of finding high‐density areas was highest at gravel contents of 15% or more. Following substrate, depth was generally the most important predictor for both species. In this study, the optimal depth of the two species was ~3–3.5 and 0.5 m for *O. edulis* and *M. gigas,* respectively. It is, however, worth mentioning that *M. gigas* also extensively colonize habitats at depths shallower than 0.5 m (Strand et al., 2012), which were not sampled here. Therefore, this study only partly accounts for the *M. gigas* distribution. *O*. *edulis* on the other hand does not occur at shallower depths than 0.5 m. Similarly, <0.2% were found at depths below 7 m, and while no samples were taken deeper than 10 m, we are not aware of any Swedish data on occurrence in deeper areas in contrast to reports from other European coasts (Kerckhof et al., 2018; Olsen, 1883; Smaal et al., 2015 and references therein).

Despite differences in optimal depth distribution between the native *O. edulis* and the invasive *M. gigas* significant overlap was observed in geographic location and depth distribution as well as for other habitat preferences, for example, substrate, exposure, and salinity. This suggests that there is a potential for various types of ecological interactions among the two species. The nature of the interactions between *O. edulis* and *M. gigas* are, however, poorly understood and is suggested to range from negative (Ruesink et al., 2005; Zwerschke et al., 2018) to facilitative (Christianen et al., 2018; Zwerschke et al., 2018), and are dependent on context. The interactive effects in Swedish waters and elsewhere remains to be evaluated.

The power and utility of various types of SDM’s in fundamental marine ecology and conservation is increasingly demonstrated (Bučas et al., 2013; Lindegarth et al., 2014; Melo‐Merino et al., 2020; Reiss et al., 2011). Recent development of extremely flexible modeling approaches and accessible software now provide powerful algorithms for fitting data and predictions. Provided that proper attention is given to avoid overfitting and violating critical assumptions, the performance of models depends largely on the quantity and quality of data, spatial and temporal resolution, and the identification of powerful predictors directly or indirectly linked to driving mechanistic processes (Guisan et al., 2007; Mannocci et al., 2017; Yates et al., 2018). In this study, it was evident that including predictors reflecting bottom substrate had the potential to improve performance and in particular specificity of high‐density *O. edulis* models. Thus, further research into the mechanistic links behind this pattern is warranted. However, due to lack of comprehensive GIS layers of substrate characteristics, this correlative link could not be used for predictive purposes.

Still, models fitted without substrate performed sufficiently well to provide useful spatial predictions for both species (i.e., AUC ≥ 0.85). This was particularly true for models of high‐density sites. Although this apparent enhanced performance may be caused by the lower prevalence of high‐density sites (Santika, 2011), it may also reflect the fact that the environmental conditions in high‐density sites are closer to optimal than those harboring occasional specimens. Furthermore, it is also likely that sampling at low densities will result in data with an increased rate of false negatives (Thorngren et al., 2017), which may affect models negatively. Irrespective of the reasons for the relative success of high‐density models, the result is encouraging considering the great importance of hotspot areas for understanding the conservation status and scope for sustainable use of *O. edulis* and the potential consequences of the invasive *M. gigas* in the region (Thorngren et al., 2019). The high sensitivity of models (>95% for high‐density models) means that they successfully identify and predict true occurrences. The slightly lower specificity (>80%) means that the occurrences and area of population hotspots are likely somewhat overestimated, particularly in Areas 3 and 4. At this point, we can only speculate about the reason for this. Further sampling and modeling are needed to verify this pattern, understand its causes, and eventually refine models. One potential explanation, however, could be that these areas have a less extensive archipelago compared with those in the north, thus affecting the retention of larvae and causing lower recruitment. In terms of locating valuable sites for conservation or monitoring efforts, this weakness is not critical.

Although there are important issues regarding the efficiency and legal implications of the different levels of protection, models and spatial predictions offer unique opportunities for coherent assessment of the geographic distribution of the two oyster species along the Swedish Skagerrak coast, which have not been possible before. In this study, we conclude that ~6% of the important *O. edulis* hotspots are covered by the highest level of protection within the state owned KNP, 44% of the population is protected by Natura 2000, and 50% of the high‐density areas are not covered by any other protection than that provided by the fishing restrictions imposed by landowners fishing rights. Consequently, with more than 80% of the flat oyster population found in less than 7% of the sampled sites, it is of particular importance to identify, manage, and protect these specific locations for the viability of the population. Additionally, even though the OSPAR member states are expected to protect, conserve, and expand the populations of flat oyster beds, the actual protection offered by Natura 2000 is debatable considering the unregulated harvest that is conducted in the areas. Although the overall fishing pressure is currently low, there are no guarantees against complete removal of local populations following an agreement with a landowner under the current Swedish regulations. Thorngren et al. (2019) estimated that the yearly harvest of *O. edulis*, mainly collected by hand, is <1% of the adult population in Sweden which is well within the limit of harvest (~5%) to still sustain a viable oyster population (Lown et al., 2020). Thus, the overall level of exploitation appears to be largely sustainable, although the status of individual oyster beds still needs to be protected from overfishing, anchoring, and other potential causes of degradation as destruction and loss of dense beds may cause severe and irreversible damage or slow recovery (Berghahn & Ruth, 2005; Eno et al., 2013), both as a consequence of reduced availability of settlement substrate and reduced broodstock densities (Guy et al., 2018; Korringa, 1952).

Finally, because the Swedish legal system does not distinguish between the two oyster species, the invasive *M. gigas* in theory is covered by the same level of protection as the native *O. edulis*. The observed data and the models suggest that high‐density areas of the two species often overlap and consequently the level of protection for *M. gigas* is similar to that of *O. edulis*. Approximately 50% of the high‐density areas for *M. gigas* coincide with areas that are also hotspots for *O. edulis*. The substantial overlap in high‐density areas means that measures implemented to reduce the invasive *M. gigas* must not jeopardize the integrity of co‐occurring populations and valuable habitats for *O. edulis* and, importantly, highlights the need to specify separate management objectives for the native and non‐native oysters.

## 5 CONCLUSION

We believe that this study provides an important contribution of knowledge regarding the current conservation status and future enhancements of management actions to protect the native flat oysters in Sweden. In particular, it provides new quantitative knowledge about environmental requirements, extent of habitats, and overlap among the two species. Furthermore, the focus on identifying hotspots is particularly useful for future efforts to monitor, protect, and restore particularly valuable sites for the Swedish native oyster population, and because this is one of few relatively intact populations in Europe, the importance of the findings is also of wider significance. Finally, from a more general perspective, we believe that the study demonstrates a coherent and generally applicable approach involving planning and execution of a cost‐efficient sampling program, modeling, and spatial prediction of habitat‐forming species of high conservation relevance over extensive spatial scales. At large scales and at depths not easily observed from surface, representative sampling and empirical modeling approaches such as this one not only provide a robust assessment of population, but may in fact be the only realistic option. In spatially less extensive areas, however, a complete inventory may be possible (although quantitative estimates abundance probably would require subsampling). Considering the accelerating threats of habitat destruction, climate change, and invasive non‐native species in the marine environment, the need for such approaches is likely to be increasingly urgent also in a wider context (Airoldi & Beck, 2007; Beck et al., 2011; Gerovasileiou et al., 2019; Lotze et al., 2006; Ruesink et al., 2005).

## CONFLICT OF INTEREST

The authors declare that the research was conducted in the absence of any commercial or financial relationships that could be construed as a potential conflict of interest.

## AUTHOR CONTRIBUTION

**Per Bergström:** Conceptualization (equal); Formal analysis (equal); Methodology (equal); Writing‐original draft (equal); Writing‐review & editing (equal). **Linnea Thorngren:** Conceptualization (equal); Data curation (lead); Funding acquisition (supporting); Methodology (equal); Writing‐original draft (equal); Writing‐review & editing (equal). **Åsa Strand:** Writing‐original draft (supporting); Writing‐review & editing (equal). **Mats Lindegarth:** Conceptualization (equal); Formal analysis (equal); Funding acquisition (lead); Methodology (equal); Resources (lead); Writing‐original draft (equal); Writing‐review & editing (equal).

## Data Availability

All data from Thorngren et al. (2019) are available through the Open Science Framework https://osf.io/jgpxw/?view_only=d070b45802a4426da028efffde3d0f76. Additional data used for modeling is available through the Open Science Framework https://osf.io/3agqp/?view_only=f2287b4c2cb041aeaca04cf66c560103.
